# Automating aortic cross-sectional measurement of 3D aorta models

**DOI:** 10.1117/1.JMI.11.3.034503

**Published:** 2024-05-29

**Authors:** Matthew Bramlet, Salman Mohamadi, Jayishnu Srinivas, Tehan Dassanayaka, Tafara Okammor, Mark Shadden, Bradley P. Sutton

**Affiliations:** a University of Illinois College of Medicine at Peoria, Pediatric Cardiology, Peoria, Illinois, United States; b University of Illinois Urbana Champaign, Bioengineering, Champaign, Illinois, United States; c University of Illinois College of Medicine Peoria, Peoria, Illinois, United States; d OSF HealthCare, Peoria, Illinois, United States

**Keywords:** three-dimensional modeling, automated segmentation, aortic aneurysm

## Abstract

**Purpose:**

Aortic dissection carries a mortality as high as 50%, but surgical palliation is also fraught with morbidity risks of stroke or paralysis. As such, a significant focus of medical decision making is on longitudinal aortic diameters. We hypothesize that three-dimensional (3D) modeling affords a more efficient methodology toward automated longitudinal aortic measurement. The first step is to automate the measurement of manually segmented 3D models of the aorta. We developed and validated an algorithm to analyze a 3D segmented aorta and output the maximum dimension of minimum cross-sectional areas in a stepwise progression from the diaphragm to the aortic root. Accordingly, the goal is to assess the diagnostic validity of the 3D modeling measurement as a substitute for existing 2D measurements.

**Approach:**

From January 2021 to June 2022, 66 3D non-contrast steady-state free precession magnetic resonance images of aortic pathology with clinical aortic measurements were identified; 3D aorta models were manually segmented. A novel mathematical algorithm was applied to each model to generate maximal aortic diameters from the diaphragm to the root, which were then correlated to clinical measurements.

**Results:**

With a 76% success rate, we analyzed the resulting 50 3D aortic models utilizing the automated measurement tool. There was an excellent correlation between the automated measurement and the clinical measurement. The intra-class correlation coefficient and p-value for each of the nine measured locations of the aorta were as follows: sinus of valsalva, 0.99, <0.001; sino-tubular junction, 0.89, <0.001; ascending aorta, 0.97, <0.001; brachiocephalic artery, 0.96, <0.001; transverse segment 1, 0.89, <0.001; transverse segment 2, 0.93, <0.001; isthmus region, 0.92, <0.001; descending aorta, 0.96, <0.001; and aorta at diaphragm, 0.3, <0.001.

**Conclusions:**

Automating diagnostic measurements that appease clinical confidence is a critical first step in a fully automated process. This tool demonstrates excellent correlation between measurements derived from manually segmented 3D models and the clinical measurements, laying the foundation for transitioning analytic methodologies from 2D to 3D.

## 1 Introduction

### 1.1 Background

Marfan syndrome, Turner syndrome, and other causes of acute aortic syndrome have significant morbidity and mortality from aortic dissection with perioperative mortality as high as 80% for emergency surgery.^1^ Marfan syndrome is well studied, and recommendations for surgical aortoplasty are widely accepted.^2^ But non-Marfan syndrome prophylactic aortoplasty recommendations remain varied. Acute aortic dissection has a significant risk of mortality,^3^^,^^4^ yet prophylactic aortoplasy is not without mortality risk, which is as high as 39% in certain syndromes.^5^

Longitudinal surveillance of the aortic aneurysm dimension remains the primary metric for medical decision making. The maximal diameter of the smallest cross-sectional area, i.e., the maximum diameter of the aorta in the plane perpendicular to the travel of the aortic arch, is the diagnostic measure that is recorded, tracked longitudinally, and compared to population normative measures. The primary reason for finding the maximum diameter is that we need to find the regions on the wall of the aorta that show the greatest risk of dissection. Cardiac MR imaging (CMR) is the preferred method by generating diagnostic resolution three-dimensional (3D) datasets and allowing for multi-planar analysis while avoiding repeated exposure to radiation or iodinated contrast. Advanced CMR techniques also now allow for non-contrast studies without the need for gadolinium, enabling CMR for those with renal impairment.^1^^,^^6^^,^^7^

### 1.2 Main Goal of the Work

Each individual patient exhibits a unique aneurysm architecture that may sit beyond standardized measurement practices, and current image analysis remains a manual process with variability (and potential error) between institution, scanning technique, and inter-observer, as well as intra-observer variability.^1^ Considering that the primary metric for a medical decision is patient-specific longitudinal surveillance of maximal aortic diameters analyzed against standardized recommendations,^1^ we hypothesize that there is a significant need to transition from two-dimensional (2D) slice by slice analysis to automated 3D aorta analysis. This project seeks to accomplish two goals. First, we create an automated tool to generate stepwise measurements of 3D aortas progressing along the centerline of the aorta; second, we demonstrate excellent correlation between the automated measurement of the manually segmented 3D model and the clinical measurement derived from standard 2D image analysis.

## 2 Approach

### 2.1 Case Selection

With IRB approval, between January 2021 and June 2022, 66 CMR congenital cardiac cases in which aortopathy was present and standard clinical measurements of the aorta were recorded were selected. Cases were acquired at a single institution (OSF HealthCare, Children’s Hospital of Illinois) by a single cardiac imager (MB). 56% were male with the age ranging between 4 and 58 years. Diagnoses were diverse and included: Marfan syndrome; Turner syndrome; intracardiac defects such as ventricular septal defects, double outlet right ventricle, tetralogy of Fallot, and truncus arteriosus (TA); D-transposition of the great arteries; and aortic valve pathologies.

### 2.2 Image Acquisition and Standard Clinical Measurement

All images were acquired on a GE Optima MR450w 1.5T MRI system. The imaging sequences were derived utilizing navigator and cardiac gated (all obtained in diastole) 3D steady-state free precession (SSFP) protocols of cardiac anatomy in axial slices between 1.2 and 1.5 mm isotropic.

A multi-planar reconstruction (MPR) and measurement software, TeraRecon, Inc., was utilized to generate clinical measurements. The software allows for 2D, semi-automated inner edge detection to create a region of interest of the aortic cross-section. The maximum and minimum diameters, area, and circumference are output. All measurements were obtained by a single cardiac imager (MB).

**Fig. 1:**
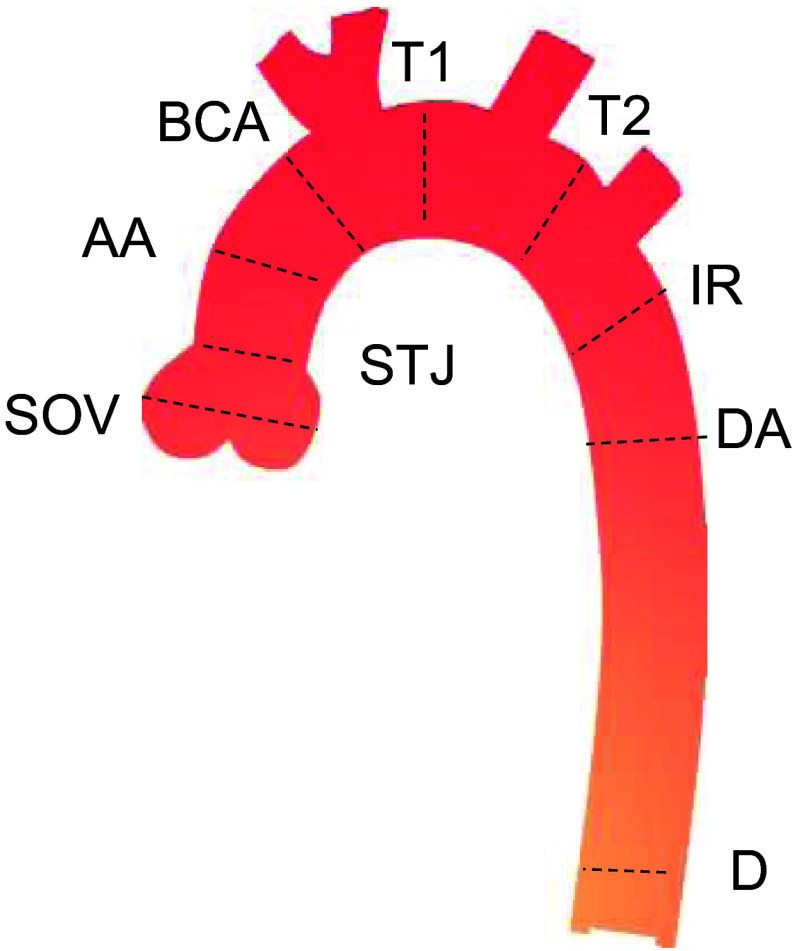
Schematic of the aortic arch with the nine standardized planes of measurement identified. These are the standard regions at which maximal diameters are obtained clinically.

As is the standard of care, nine standardized locations were used to generate clinical measurements of the following sites: sinus of valsalva (SOV), sino-tubular junction (STJ), ascending aorta (AA), before brachiocephalic arteries (BCA), transverse segment 1 (T1), transverse segment 2 (T2), isthmus region (IR), proximal descending aorta (DA), and aorta at diaphragm (D).^8^ These locations are shown schematically on an aortic arch in Fig. 1.

**Fig. 2:**
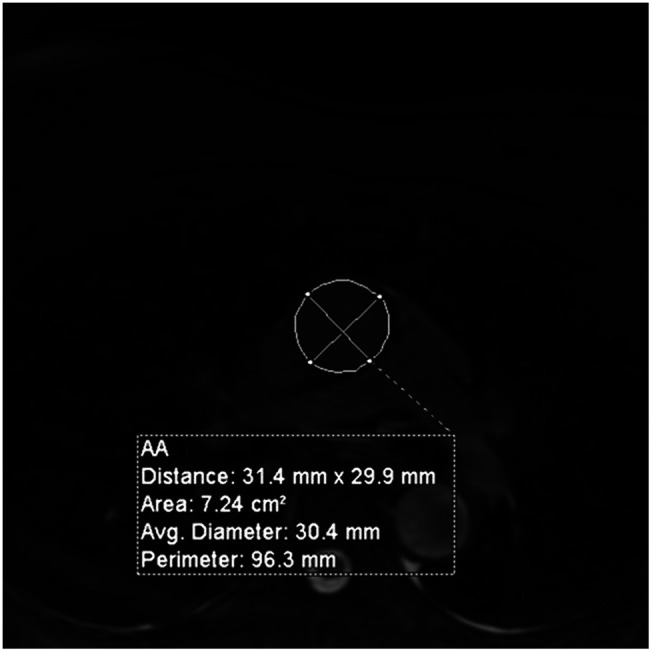
Example of a clinical measurement obtained on a patient at the level of the right pulmonary artery. The diameters are drawn across the aorta, and the maximal diameter is used in the clinical measurement.

The MPR measurement tool was utilized to attempt to acquire a perpendicular slice to the aorta, generating a minimum cross-sectional area and contingent maximal diameter clinical measurement at each location. With the measurement plane defined, the maximum diameter is the primary value utilized for clinical analysis. This value can then be compared longitudinally within a patient or across patients with standardized values.^8^ In complex aortic root arrangements, the ability to obtain an accurate minimum cross-sectional area can be quite subjective. The clinician time to complete the measurement process, transfer, and record to the electronic medical record typically takes 15 to 30 min. A representative example of a measurement of the AA at the level of the right pulmonary artery is shown in Fig. 2.

Clinical emphasis is focused on the region of greatest diameter. In some patients, artifacts or a lack of anatomical markers (i.e., a common trunk of the brachiocephalic artery and left carotid artery eliminates transverse segment 1) may result in some measurements not being recorded.

### 2.3 3D Model Generation

**Fig. 3:**
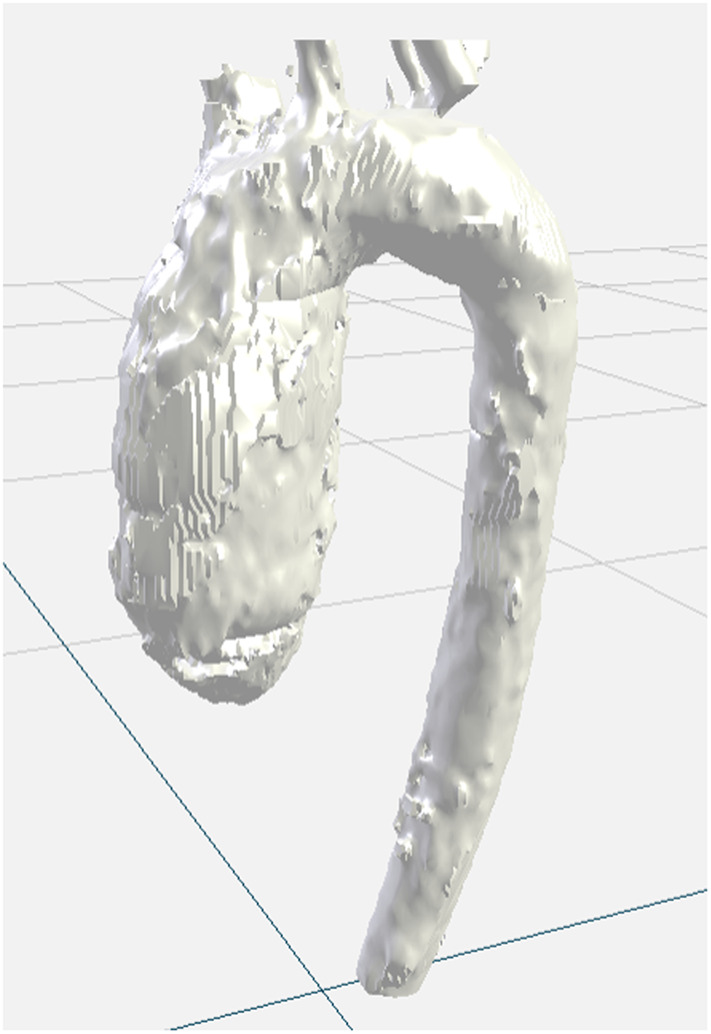
Example 3D model derived from manual segmentation of the aortic arch by trained raters.

To enable an automatic measurement, we first need to build 3D models of the aortic arch. For each case, the 3D SSFP sequence was de-identified and imported into a medical segmentation software, Mimics Innovation Suite, by Materialise, Inc. The segmentation team consists of trained anatomists that regularly perform segmentation of various structures to form presurgical planning models. The team segmented the aorta using available tools within the Mimics software to achieve an inner-edge to inner-edge segmentation slice by slice from left ventricular outflow tract (to ensure inclusion of the aortic valve annulus) through the aorta down to the level of the diaphragm. A simple thresholding of the blood pool signal intensity was used as an initialization. Once thresholding limits were determined to capture most of the aorta, a region of interest was manually drawn around the aorta from start to finish and an interpolation tool then applied the threshold to the selected aorta. Due to the intrinsic intensity variation in MRI due to receiver coil sensitivity patterns, a global threshold is not sufficient for segmentation, and manual adjustments of the mask are necessary in different regions of the aorta. No smoothing was performed. Time to manually segment each aorta was estimated at around 20 to 40 min. A 3D model was generated for each case and exported as a .stl file. An example of a segmented aorta is shown in Fig. 3.

### 2.4 Automating Measurement of the 3D Models

With a 3D model of the aorta, the next step is to generate diagnostic measures along the aortic arch. In this workflow, we generate an over-complete set of measurements of maximal diameter of minimal cross-sectional area and have the clinician select the measure at the regions they wish to take the measurement. The process of making the diameter measurements involves several steps: (1) identification of the centerline of the aorta along with basic image processing steps to adjust the centerline to ensure that it is perpendicular to the main axis of aorta and (2) determination of the maximum diameter in that cross-section. Therefore, we first describe an algorithm for discretely estimating the centerline, point-by-point along the aortic arch.

#### 2.4.1 Algorithm description

Several previous tools exist to estimate the centerline from a segmented vessel, a critical component of our automated measurement. One popular tool is the Vascular Modeling Toolkit (VMTK), by which segmented vessels are filled with a series of spheres that are grown until they reach the maximum extent of the vessel.^9^ By connecting the centers of these spheres, a centerline in a vessel can be created through the vessels, and the maximum diameter of the sphere can represent the maximum dimension of the aorta. In this work, we define the centerline and maximum diameter in a manner that is driven by the manual clinical process for determination of the centerline. We find the minimal cross-sectional area as we take steps along the aortic arch, determining the planes perpendicular to the arch similar to how the clinician would do it themselves. This approach is similar to the approach of Bondesson et al., in which a plane is defined as perpendicular to the aorta at each location along the aorta, defined by the minimal cross-sectional area.^10^ They further fit the centerline with a cubic polynomial spline; we propose that maintaining the original centerline as its exact shape is not important to our primary output of measures of the maximal diameter of the aorta.

We provide a brief description of our algorithm for tracking the centerline and making maximum diameter measures at various points along the aorta. To start, we take two adjacent straight-axial slices at the very inferior portion of the imaging volume. In this region, the aorta is running very nearly vertical, and this is used as an initialization of the centerline. For each of these two slices, we calculate the center of mass of the aortic arch and identify that center point in each slice. Connecting those two points together forms the initialization of the center line for tracking.

**Fig. 4:**
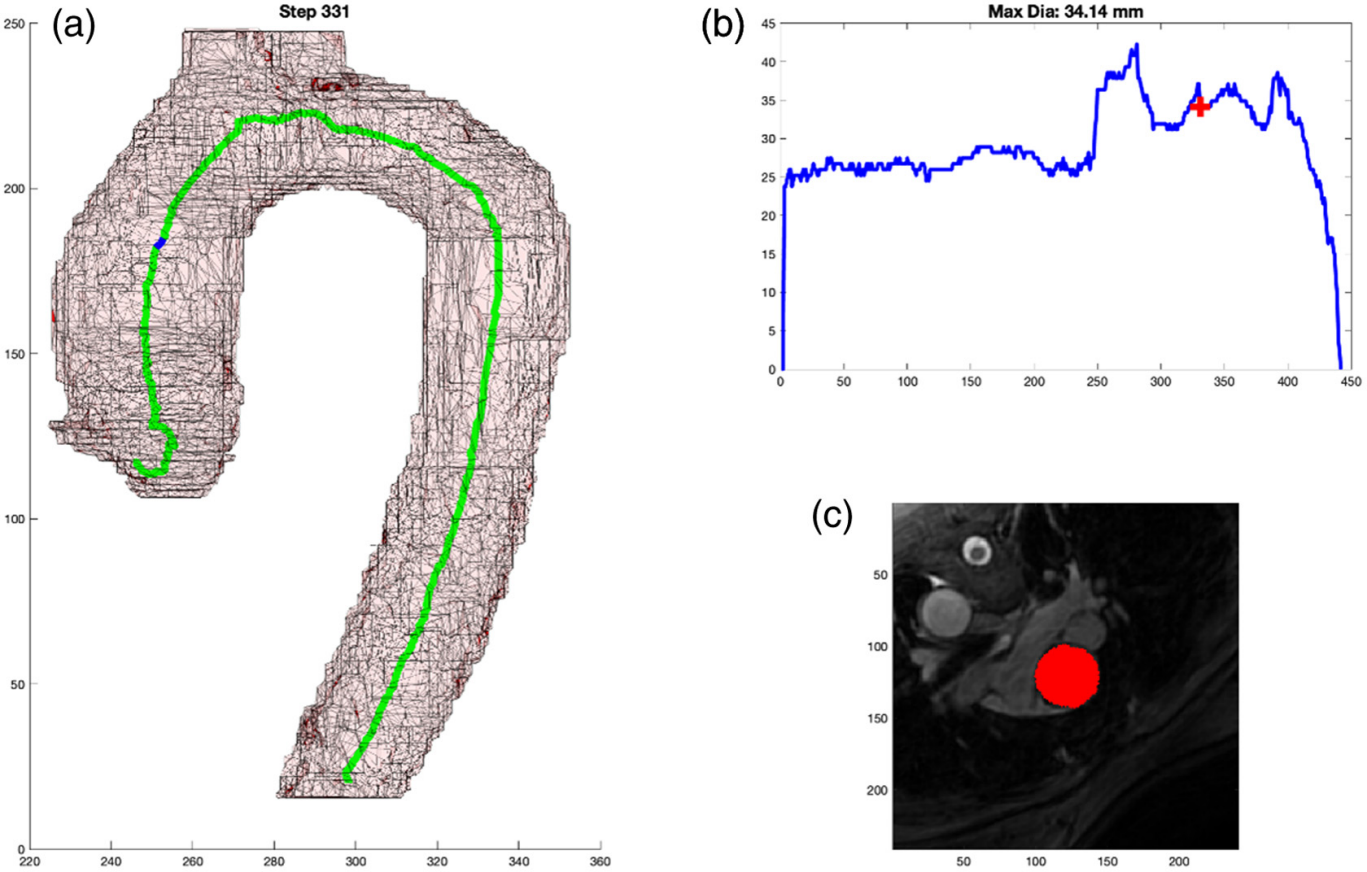
(a) 2D representation of 3D model with marker at the location of the cross-sectional measurement. (b) Graph of aortic diameter automated measures from the diaphragm to the root. (c) Mask of the cross-sectional area measured by the automated process that is overlayed on the MPR MR image.

After the initialization, we proceed to track the center line of the aortic arch, proceeding superiorly, using the 3D mask of the aortic arch model. At each step, we perform several processes: (1) propagate the arch by extrapolating a fixed step size based on the previous two points of the aortic arch; we typically choose to take a step size equivalent to the smallest imaging dimension, which is <1  mm. (2) With this point identified and using the angle of the step in part (1), we search over varied angulations (±30  deg) to find the minimal cross-sectional area. We call this process “wobbling.” We wobble until the minimal cross-sectional area is identified, and that becomes the new perpendicular cross-section for the aorta at the current step. To determine the cross-sectional area of each newly sampled 2D slice, we use the 3D mask and iteratively grow the mask to ensure that only connected pixels in the 2D plane are included in the cross-sectional area measurement. This is necessary at superior slices through the aorta as multiple instances of the aortic mask may intersect the perpendicular plane. (3) Then, we calculate the center of mass through the cross-section of the aortic mask to identify the new center line point at that cross-section. (4) We can now repeat back to process (1) for the next step in the centerline. This process tracks the aortic arch centerline well, as shown in Fig. 4 (Algorithm 1).

**Algorithm 1:** Centerline tracking and maximum diameter measurement along the aorta.

**Input:**
- Aortic arch data comprising cross-sectional images
- Initial starting point at the inferior point of the aortic arch where it passes through the diaphragm
**Output:**
- Centerline coordinates at various points along the aorta
- Maximum diameter measurements at corresponding points
- Cross-sectional images with mask overlayed to demonstrate the plane of measurement
Stepwise description:
1. Initialize the starting point at the inferior point of the aortic arch and set the current plane angle to 0, finding the center of mass of the aortic mask at two inferior axial slices.
2. Propagate the centerline by taking a step in the direction indicated by the two preceding centerline points.
3. At that point, find the angle that minimizes the cross-sectional area: <3.1> - Perform a search by incrementally varying the plane angle. <3.2> - For each angle, calculate the cross-sectional area. <3.3> - Identify the angle that results in the minimum area.
4. Define a new plane using this angle that minimizes the cross-sectional area.
5. Calculate the center of mass of the aortic mask within the new plane. This becomes the new centerline point.
6. Calculate the maximal diameter of the aorta from this plane. Create output visualizations.
7. With the new centerline updated, repeat back to step 2 to calculate the next point in the centerline. Stop when the centerline exits the aortic arch mask.

With the centerline tracked and the perpendicular cross-sectional area defined relative to the arch, we take the maximal diameter measurements at each location and create visualizations to enable the clinician to verify the accuracy of the method and to pick the region at which the measure was obtained. Note that this can be done coincident with process 3 at each step of centerline tracking to avoid resampling the 3D model and image multiple times. The process results in a series of stepwise measurements from the diaphragm to the aortic root, along with images overlaying the manual mask on top of the image and indicating where the measurement was obtained.

In most instances, there were 300 to 400 steps to track from beginning to end of the aortic arch, with each step being on the order of the smallest dimension of the imaging pixels, i.e., <1  mm. The entire centerline tracking algorithm completes automatically in under an hour for each patient running on a standard engineering workstation in MATLAB. This compares to 15 to 30 min of clinical intervention in the standard historical measures, which includes making the measurements and documenting the measurements on the MPR slices at each of the nine measurement locations.

### 2.5 Output Format

As shown in Fig. 4, there are three components to the output. Figure 4(a) shows the centerline generated as well as a blue dot to indicate the location of the current step as the centerline propagates through the 3D arch model. Figure 4(b) shows the graphical representation of the measurement along the continuum with a red cross indicating the corresponding location for that step. And Fig. 4(c) shows the MPR cross-section of the DICOM image from which the minimum cross-sectional area was measured along with the red mask over the aorta at that location.

### 2.6 Selecting 3D Values for Correlation

**Fig. 5:**
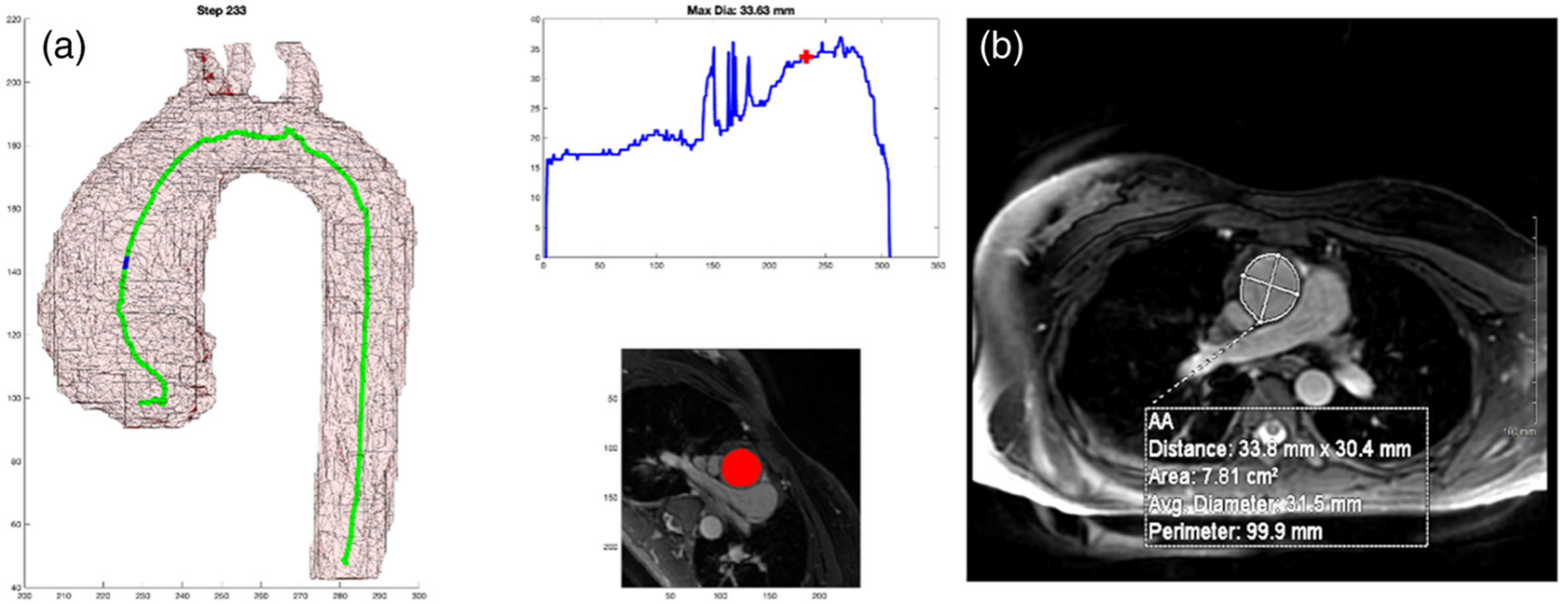
(a) Example output of the aortic arch centerline tracking algorithm compared to (b) the clinical output measure viewed in DICOM.

For each of the 50 cases, a clinical subject matter expert (MB) reviewed, side by side, the clinical measurement reference image and attempted to select (from the ∼400 frames generated, Fig. 4) the frame where the automated DICOM image with the mask most closely paralleled the clinical DICOM image where the clinical measure was obtained. This was done by examining the anatomy around the arch along with looking at the 3D model of the arch and the blue point in it. This was repeated for each case and at each of the nine locations along the aorta. Figure 5 shows an example of the algorithm output in Fig. 5(a) and the clinical measure documented for the patient in Fig. 5(b).

### 2.7 Statistical Analysis

Single, two-way intraclass correlation (ICC) analysis was performed to measure the level of agreement between the clinical 2D values and the values derived automatically from the 3D models.

## 3 Results

### 3.1 Success and Failure of Automated Measurements

**Fig. 6:**
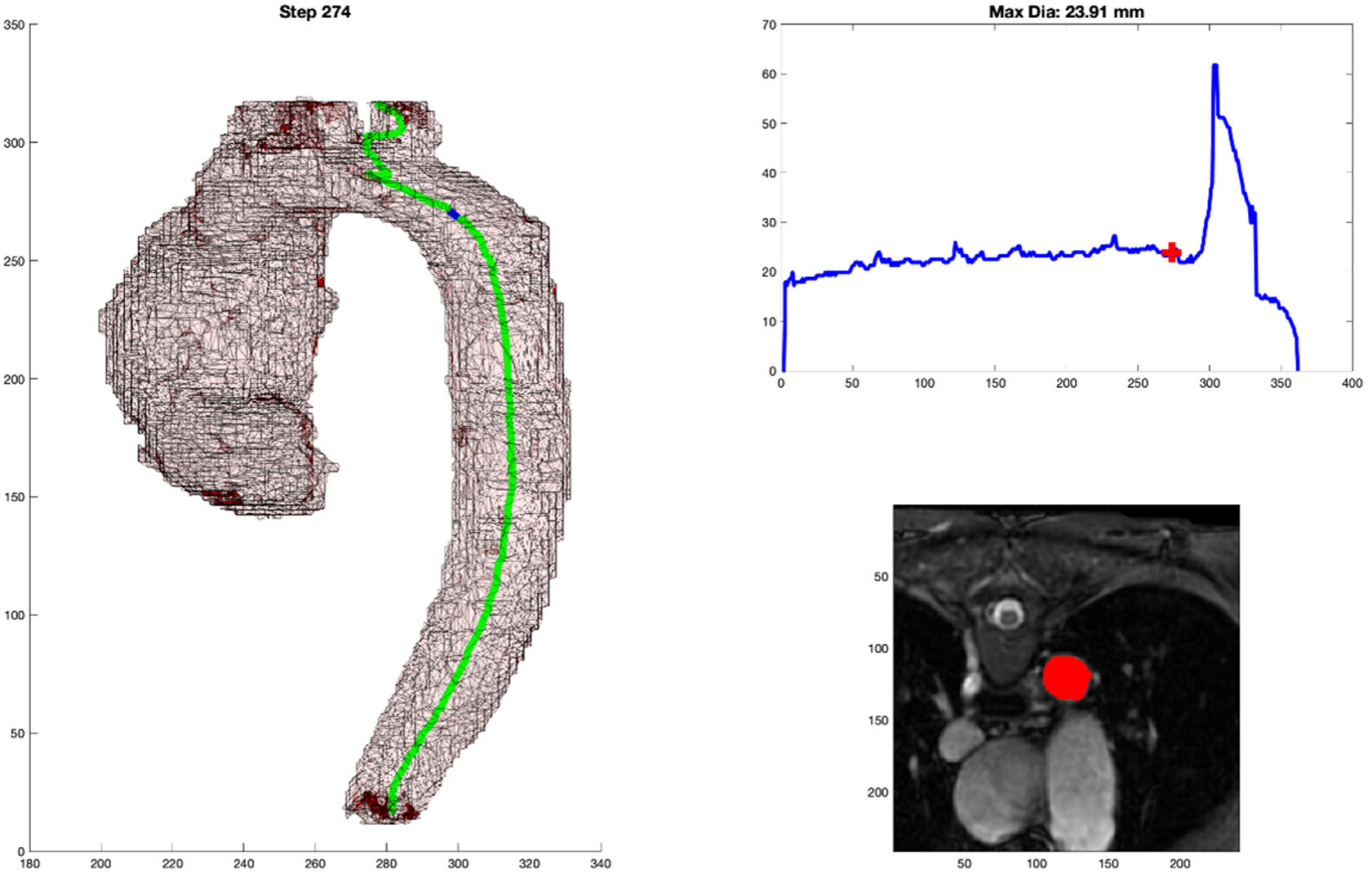
Failure of centerline to traverse the aortic arch, instead, exiting up the left subclavian artery.

The centerline detection and maximum measurement algorithm successfully output stepwise frames from the diaphragm to the aortic root in 76% of the cases. However, in 24% of cases sampled, the centerline failed to traverse the aortic arch or was interrupted by segmentation errors related to an artifact. In most instances, the centerline redirected itself up the left subclavian artery. An example of this is shown in Fig. 6.

**Fig. 7:**
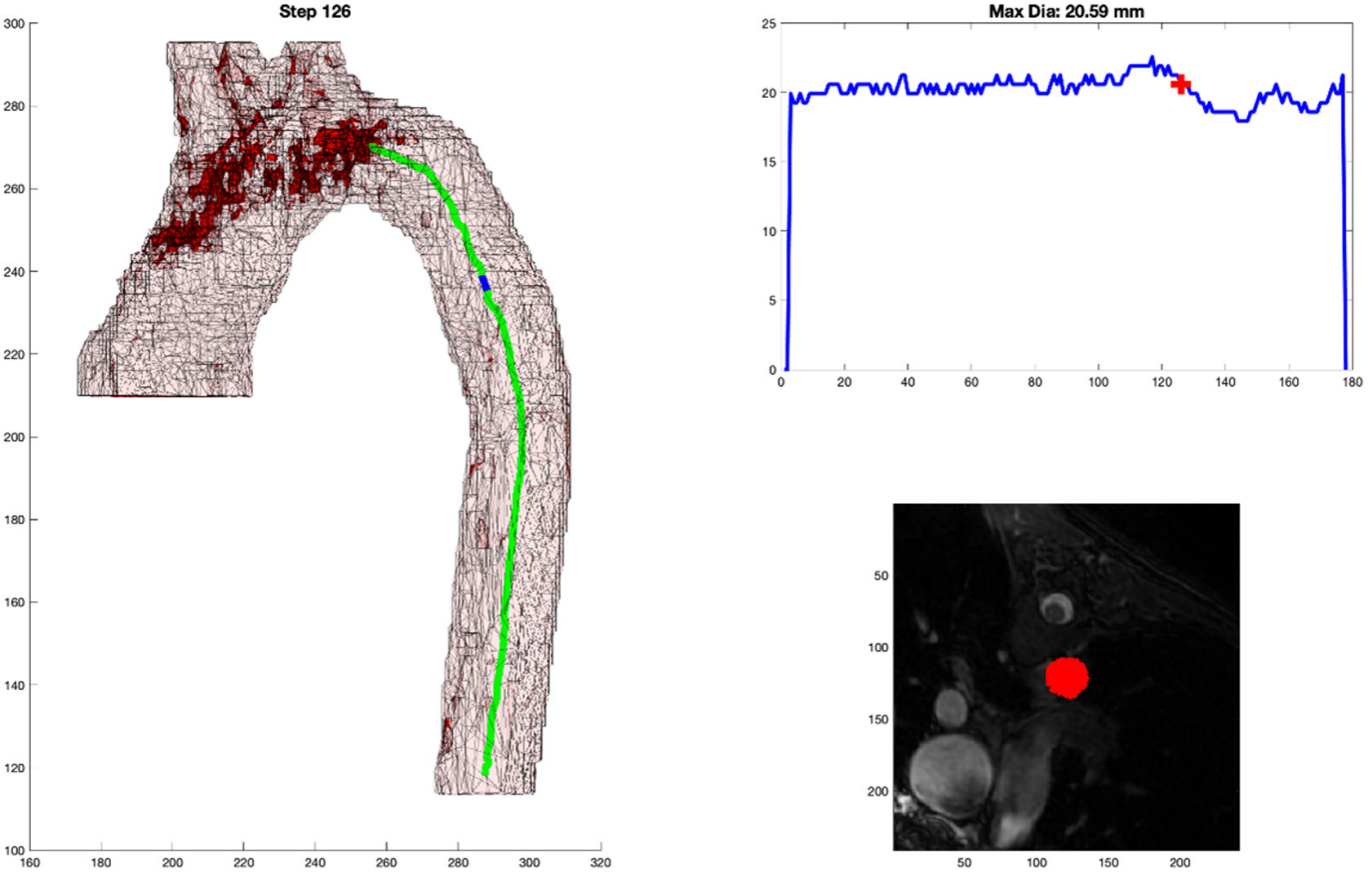
Failure of centerline to traverse the aortic arch due to a segmentation error from a poor signal intensity in the AA. The dark red patches represent cavitations within the 3D model.

In a few cases, the tracking algorithm was interrupted due to pockets of un-segmented aorta within the aortic lumen. The segmentation errors were due to a lack of signal within the lumen of the aorta. These cavitations confused the centerline detection and caused it to turn back on itself or stop forward progress. An example is shown in Fig. 7.

### 3.2 Interclass Correlation Between the Two Groups

ICC analysis assessed the level of agreement between the standard clinical and automated measurements for each of the nine measurement locations (if available). The results indicate an excellent level of agreement at each site; see Table 1.

**Table 1:** ICC values between automated measurement and historical clinical measurement across the samples in this study. Note that the number of samples varies across the different measures due to the absence of clinical measures in regions of complex anatomy.

	ICC Coef.	95% C.I.	p-value	N
SOV	0.99	0.98 to 0.99	<0.001	49
STJ	0.89	0.58 to 0.96	<0.001	50
AA	0.97	0.89 to 0.99	<0.001	50
BCA	0.96	0.84 to 0.98	<0.001	50
T1	0.89	0.77 to 0.94	<0.001	35
T2	0.93	0.84 to 0.96	<0.001	48
IR	0.92	0.79 to 0.96	<0.001	49
DA	0.96	0.87 to 0.98	<0.001	49
D	0.93	0.80 to 0.97	<0.001	47

**Fig. 8:**
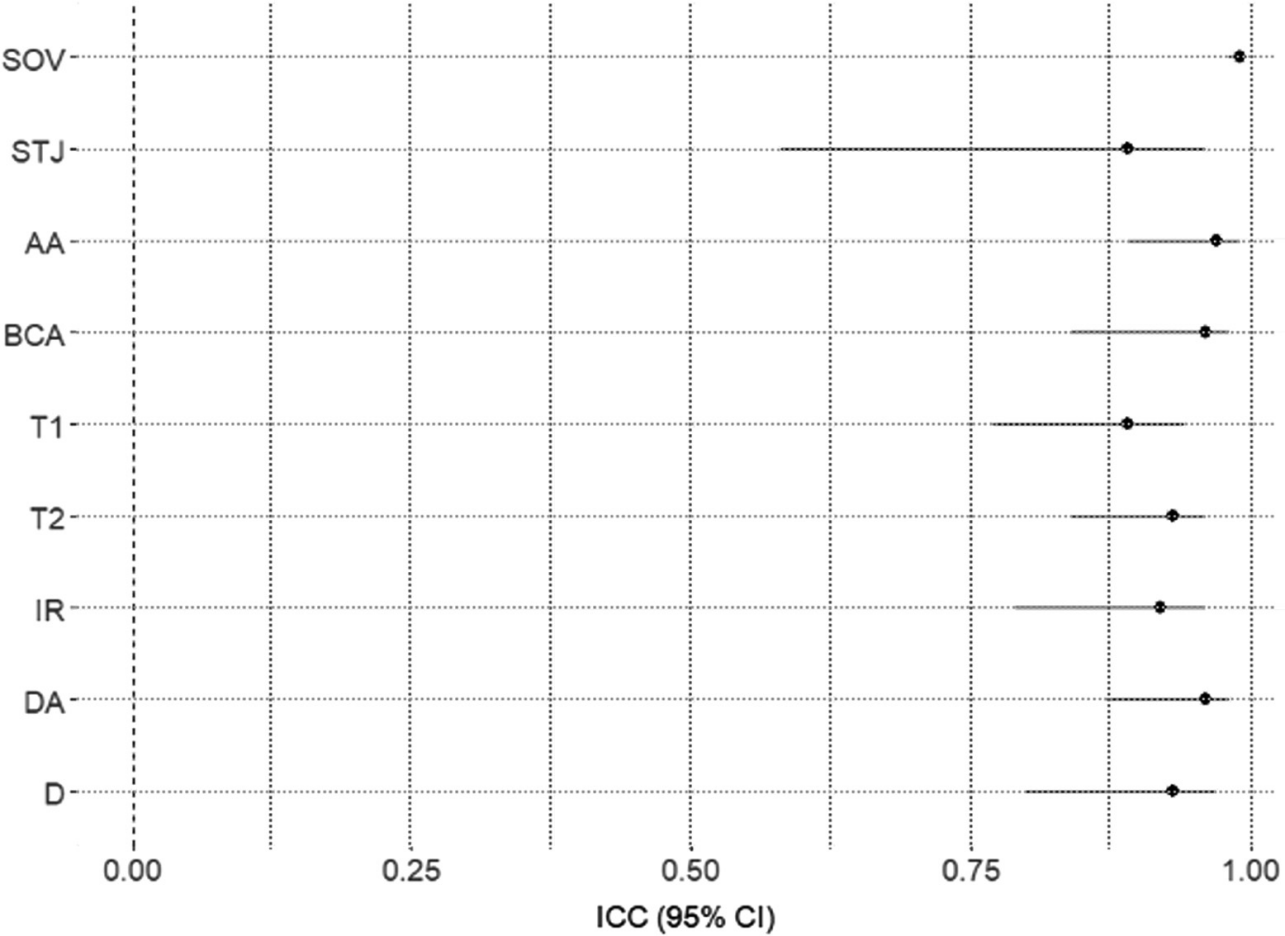
Forest plot of ICC between standard manual clinical measures and the automated measures based on the centerline tracking algorithm.

Three of the locations (STJ, AA, and BCA) had comparable values for all 50 cases. The SOV had one case in which no clinical measurement was obtainable due to an artifact from an artificial aortic valve. The transverse arch, especially T1, had the fewest measurements due to anatomic variations as pointed out in Sec. 2.2. The clinical region of interest where the largest diameter occurred was adequately captured in each case. This could also be a function of the clinical image acquisition ensuring adequate resolution through the region of interest at the time of the exam. A forest plot, shown in Fig. 8, showcases the excellent consistency and precision between the automated and 2D clinical measures throughout the aorta with the STJ being the only outlier. This is likely due to the blurring of the border between the sinuses and the tubular structure of the aorta that occurs as it dilates, making it difficult to correctly select the exact location of the junction.

**Fig. 9:**
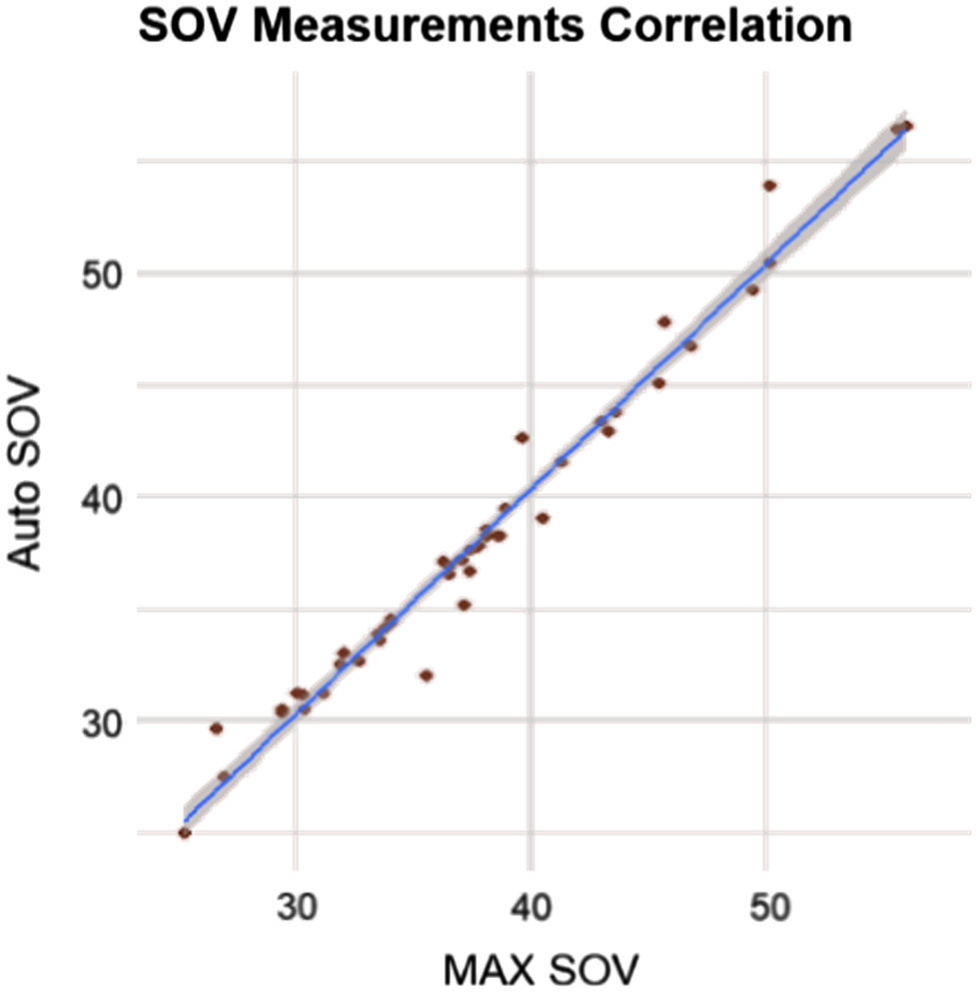
Scatter plot of automated versus clinical measures in the SOV, showing high agreement.

**Fig. 10:**
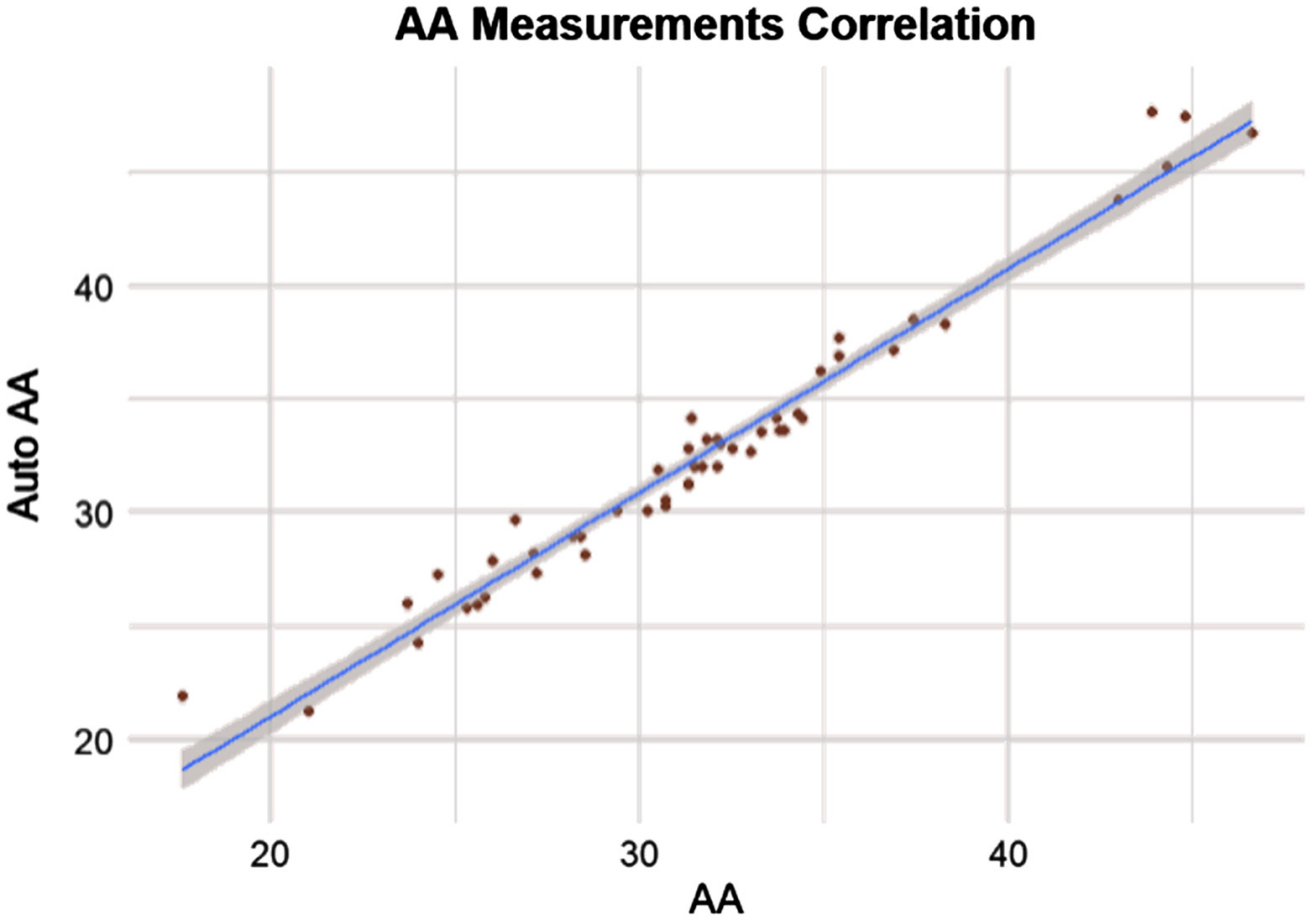
Scatter plot of automated versus clinical measures in the AA, showing high agreement.

From a clinical perspective, only the largest diameter is of primary concern among all nine standard locations, as this drives the decision for aortic root replacement. The two primary sites for this maximal aneurysmal dilation are the SOV and the AA. These two sites represent the best correlations throughout the aorta, as represented by the scatterplots in Figs. 9 and 10.

## 4 Discussion

### 4.1 Clinical Validation

These findings validate the initial step of transitioning clinical measurements of aortic arch diameters from manual measures to automated measures leveraging the full 3D model. The excellent correlation between methodologies proves that a 3D segmented model of an aorta can accurately act as a surrogate of 2D MPR perpendicular sliced imaging. This finding lays the foundation and validates future methodologies that intend to use segmented 3D models as training datasets for machine learning toward automated segmentation. Without first proving that the 3D models correlate to clinically derived measurements, there could be introduction of a weak link in the chain of development that could result in a lack of trust of future work. Clinical experts will rely on this proof to apply validity to future work revolving around 3D segmented models of aortic arch analysis.

### 4.2 Importance of Output Format

As shown in Fig. 4 and referenced in Sec. 2.5, the output format was very deliberate. It is the job of the clinician to critically evaluate new methods of measurement. By including the three key components: 3D model with centerline and marker, graphical representation of measurement with localizing marker, and DICOM MPR image with mask overlay of aorta; the clinician has all of the necessary information needed to trust the measurement and its relationship to traditional methods. This output becomes even more important once automated segmentation takes the place of the manual segmentation performed in this project. The 3D representation of the aorta provides a quick quality check regarding the gross segmentation process. The graph allows for a very fast analysis of the largest diameter. Incidentally, the process generates three spikes (related to perpendicular slices extending into the head and neck vessels with the maximum diameter of the cross-sectional slice extending up into the vessel); this helps to orient the user to the location. The DICOM MRP image with mask overlay is probably the most important. This output can be sent back to a standard PACS viewer where measurements can be obtained in traditional methods, which will undoubtedly be required to transition trust from historical manual measurements to fully automated tools. A clinician who can use this tool and personally validate the automated measurements will have all of the resources that they need to personally gain trust in a tool such as this.

### 4.3 Clinical Benefit of This Methodology

**Fig. 11:**
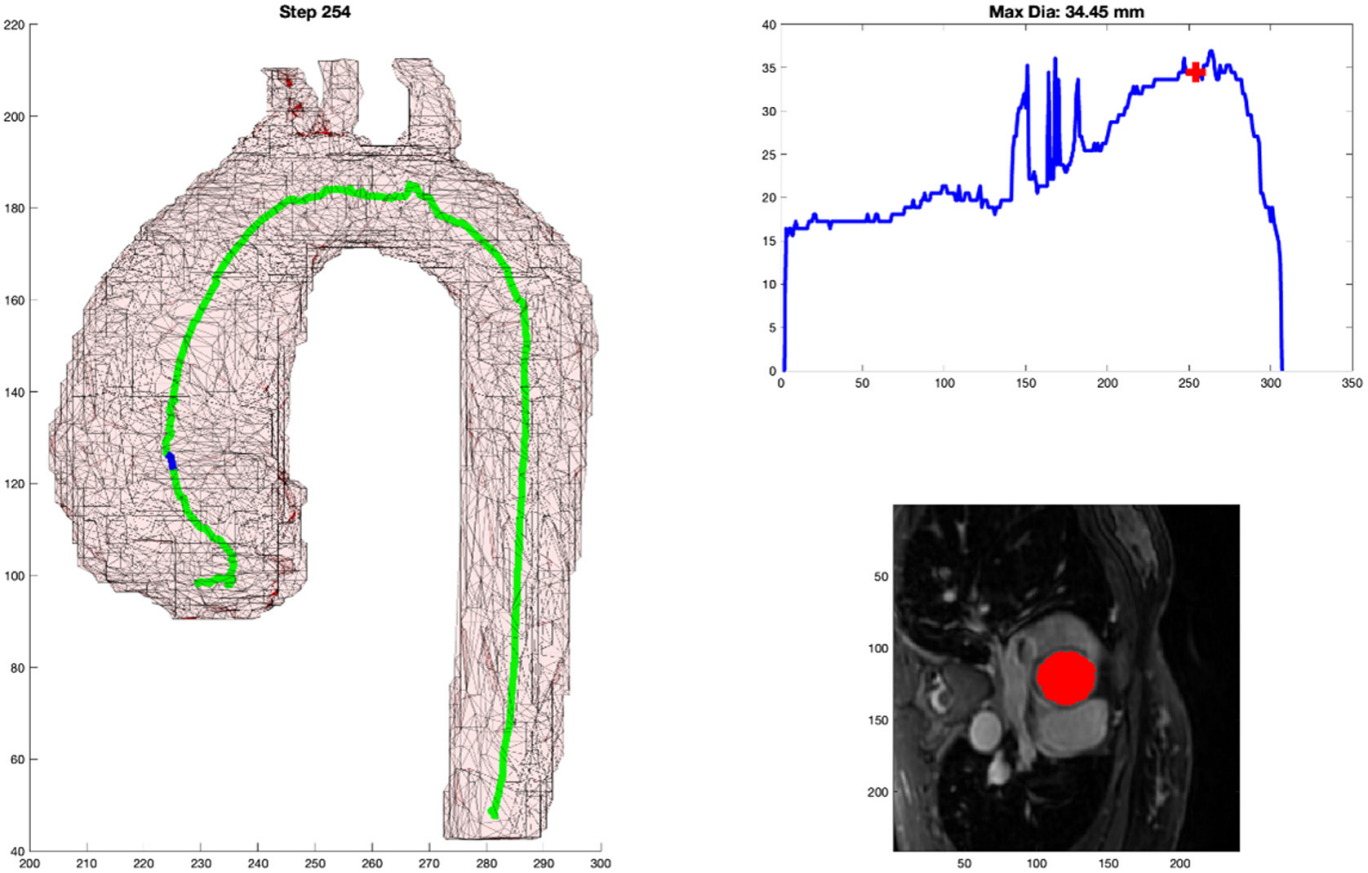
Same patient as in Fig. 5, but the automated centerline tracking identified a maximal diameter that was not at one of the standard measurement locations. The maximal diameter is of primary interest to the clinician, and this additional information from the centerline adds information for consideration in the treatment pathway.

As stated in Sec. 2.2, it can be difficult to accurately reproduce a perpendicular cut plane through an aorta with unusual morphology. An oblique plane will produce an over-estimation of the maximal dimension, which could have clinical implications. Through the mathematically derived centerline detection and minimal cross-sectional cut-plane detection, human error can be significantly reduced. In addition, rather than relying on a single slice through the aorta, a near continuous step-wise output of measurements through the aorta can help to highlight the greatest region of aneurysmal dilation unencumbered by a clinical proclivity to obtain a measurement at a specific location. In many of these cases, the parallel DICOM images would match very well, but would not represent the greatest diameter detected by the computer. In Fig. 5, the clinical measurement compared to the (automated measurement) for three of the locations were as follows: SOV: 33.6 mm (33.63 mm), STJ: 33.6 mm (33.63 mm), and AA: 33.8 mm (33.63 mm), respectively. But as shown in Fig. 11, a reliable, automated, measurement of 34.45 mm can be seen as the peak of the curve, but it occurs in a different region relative to the standard measurement planes.

When performing clinical measurements, the clinician aims to include the largest diameter of the aorta, but even with an MPR viewer, the full 3D characteristic of the aorta may not be fully visualized, and the largest region could be missed. This automated, near continuous measurement method, when combined with algorithmic certainty of identifying minimal cross-sectional areas, should dramatically improve the reliability of longitudinal surveillance of these aneurysmal aortas and improve derived medical decisions.

The automated tracking of all regions of the aorta provides a rich set of measures of the maximal diameter of the aorta across the entire anatomy. This can serve to improve the detection of longitudinal changes in a patient over time. The presence of a full set of diameter measures over the arch will provide information on these longitudinal changes at a resolution not previously achieved and will enable investigation into new measures of regional characteristics of the arch.

### 4.4 Limitations

In the results shown here, the clinical expert subjectively selected the automated image (based on gross anatomic detail) that most closely resembled the clinical image at each of the respective nine locations. Because 3D positioning of the perpendicular slice was not captured in the clinical dataset, this image matching was used as a surrogate to exact slice position. (As the angle of a slice changed slightly, the surrounding anatomy that is farthest from the center would change dramatically.) This method could potentially introduce bias in the measurement point. However, the maximal diameter measures were obtained fully automatically at every point along the aortic arch. The maximal diameter curves are finely sampled along the aorta from the automated measure and produce curves that are generally smooth in most locations. Any potential bias in the exact positioning of the measurement slice is not expected to have a significant impact on the correlations between automated and manual measurements presented here. Automated determination of the sampling plane for each of the standard clinical measures will be pursued in future work.

The failure rate of the centerline tracking remains high. As shown previously, some of these were due to incomplete segmentation in the creation of the 3D model and some were due to exiting the model due to another vessel. Both types of errors will be addressed in future work. Furthermore, in various subjects, the presence of an implant, such as an artificial valve, created significant inhomogeneous regions in the source image. The clinical imaging team performs QA immediately after scanning per standard of care protocols. If the aortic regions of interest (areas of greatest dilation) are sub-optimal for clinical review in the images, the parameters are adjusted to re-acquire a dataset adequate for clinical measurement at the time of the study. This QA process benefits the automated analysis as well as the manual clinical process. For the clinical measurements, locations impacted by these artifacts were not measured; hence, they were not included in the comparisons shown in this paper.

The manual segmentation process was rough and included some external aortic structures in some instances. These could be seen as small spikes in the aorta measurement graph. These spurious additions are not part of the aorta and can interfere in the determination of the minimal cross-sectional area in our automated measurements along with impacting the maximal diameter estimated. In previous automated segmentation work, we witnessed improvements in anatomic smoothing from the machine learned automated segmentation over the manual segmentation.^11^^,^^12^ This occurs as the machine learns the overall shape of the aorta, for which the small “spikes” are random and get averaged out. In future work, we will explore this smoothing in addition to other smoothing operations that can be applied to the model prior to centerline tracking.

### 4.5 Next Steps

We plan to continue toward a fully automated tool that will perform the following: automate segmentation of the aorta, automate centerline detection and measurement, and automate diagnosis based on 3D model pattern recognition. As an example, in many cases of bicuspid aortic valve, the fused cusps have a significantly lower cusp-cusp measurement than the two opposing cusps; this finding may allow for automated diagnosis of this condition, not to mention other diagnoses with reproducible 3D patterns.

Several previous works have implemented fully automated segmentation tools for the aortic arch, but many of the existing tools were created for CT angiography data,^13^ phase contrast,^14^ or 4D flow MRI acquisitions,^15^ whereas we focus here on contrast-free 3D cardiac MR. We use manual segmentation of the aortic arch for constructing 3D models in this work, with our main goal to show reliability of the automation of the clinical measures from a 3D model. However, for a fully automated method, we will integrate this automated segmentation in a future version of the algorithm.

Finally, with a fully automated tool, this algorithm should be able to sit within a PACS system and generate cohort specific z-scores at great scale. This will open the door for improved comparative data in a similar cohort of patients whose current z-scores are not available.

## 5 Conclusion

Automated centerline detection and measurement of manually 3D segmented aortas demonstrates excellent correlation to clinically derived measurements at the standard nine measurement locations. In addition, the detection of maximal diameters from a dense sampling of cross-sections of a full 3D representation of the aortic arch allows for more robust measures of dilation instead of focusing on just a few predefined locations. These findings lay the foundation for transitioning from 2D centric methodologies to 3D methodologies in clinical analytics.

## 6 Appendix:

**Video 1** Example of a video still image (MOV, 16.6 MB [URL: https://doi.org/10.1117/1.JMI.11.3.034503.s1]).

## Supplementary Material



## Data Availability

All data in support of the findings of this paper are available within the article. For additional data and material questions, please reach out to the corresponding author.
